# Critical Role of AdipoR1 in Regulating Th17 Cell Differentiation Through Modulation of HIF-1α-Dependent Glycolysis

**DOI:** 10.3389/fimmu.2020.02040

**Published:** 2020-08-18

**Authors:** Qian Zhang, Lei Wang, Jintao Jiang, Shiyu Lin, Aishu Luo, Pengfei Zhao, Wenfeng Tan, Miaojia Zhang

**Affiliations:** Department of Rheumatology, The First Affiliated Hospital of Nanjing Medical University, Nanjing, China

**Keywords:** AdipoR1, Th17, glycolysis, HIF-1α, AIA

## Abstract

We previously reported that adiponectin (AD) promotes naïve T cell differentiation into Th17 cells and participates in synovial inflammation and the bone erosion process in patients with rheumatoid arthritis. Here, we use a T cell lineage adiponectin receptor 1 (AdipoR1) conditional knockout model to investigate the role of AdipoR1 in Th17 differentiation. RNA-sequencing (RNA-seq) demonstrated that *AdipoR1* knockout reduced the expression of a variety of T cell related genes, with *Rorc* showing the greatest level of down-regulation. AdipoR1 deficiency inhibited Th17 cell differentiation *in vitro* and ameliorated joint inflammation in antigen-induced arthritis mice. Moreover, AdipoR1-deficent CD4^+^T cells displayed reduced Hypoxia-Inducible Factor-1α expression leading to glycolysis inhibition during naïve CD4^+^T cell differentiation into Th17 cells. We describe a novel function of AdipoR1 in regulating Th17 cell differentiation through modulating HIF-1α-dependent glycolysis.

## Introduction

Rheumatoid arthritis (RA) is a common chronic autoimmune disease that is characterized by synovial inflammation, autoantibody production, and cartilage and bone destruction (1). Though the cause of RA remains unknown, accumulating evidence indicates a critical role of Th17 cells in disease development in both experimental arthritis models and in human RA (2).

Recently, adipose tissue was recognized as an important endocrine organ. Adipose tissue plays an important role in the regulation of various physiological functions through the secretion of a number of adipokines. Adiponectin (AD) is the most abundant adipokine in the circulation, accounting for approximately 0.01% to 0.05% of total plasma proteins. Growing evidence suggests that AD plays a vital role in regulating immune and inflammatory processes (3, 4). Previously, we showed that AD treatment markedly enhances Th17 cell generation *in vivo* and *in vitro* (5). Our data provide robust evidence that AD participates in the regulation of Th17 response. However, the underlying molecular mechanism remains unclear.

Naïve T cells can differentiate into various T helper (Th) cells. During this process, metabolism reprogramming occurs to meet energy requirements and provide various indispensable substrates for T cell proliferation and differentiation. Manipulating metabolic pathways in T cells can shape their fate and function. Th17 cells mostly rely on aerobic glycolysis, a sequence of cytosolic enzymatic reactions that convert glucose into pyruvate, generating energy (6–8). In an autoimmune model of RA, inhibition of glycolysis can impact the Th17/Treg balance and reduce disease severity (9).

HIF, a heterodimer comprised of α (HIF-1α) and β (HIF-1β) subunits, is a key transcription factor that orchestrates the expression of glycolytic enzymes. HIF-1α is up-regulated under Th17-polarizing conditions and promotes glycolysis during Th17 differentiation (10). HIF-1α plays a dual role in Th17 development by directly activating Rorγt transcription and then associating with Rorγt at the IL-17A promoter to recruit p300 (11).

Our previous studies have shown that AD drives expression of HIF-1α in synovial fibroblasts. Here, we hypothesize that the AD-HIF-1α pathway contributes to regulating Th17 response in the pathogenesis of RA. AD exerts its functions by acting on its receptors, adiponectin receptor 1 (AdipoR1) and adiponectin receptor 2 (AdipoR2). AdipoR1 is highly expressed in skeletal muscle, while AdipoR2 is detected in the liver and quadriceps muscle. Previously, we have demonstrated that AD and AdiopR1 are more highly expressed in synovial tissues from patients with RA than in those from patients with osteoarthritis (4). Further, we confirmed that AdipoR1 is prominently expressed in T cells from patients with RA (unpublished data), suggesting that AdipoR1 is critically involved in synovitis and T cell response in RA.

In the present study, we generated CD4^+^T cell-specific AdipoR1 conditionally deficient mice to examine the role of AdipoR1 in Th17 cell differentiation *in vitro* and on AIA development. Our data indicate that loss of AdipoR1 reduces disease severity in AIA. Moreover, AdipoR1 -deficient T cells decrease Th17 differentiation through inhibition of HIF-1α-dependent glycolysis in T cells.

## Materials and Methods

### Mice

In order to obtain AdipoR1 lox mice, Cas9 mRNA, sgRNA and donor were co-injected into zygotes. sgRNA create DSBs (double-strand breaks) in intron 2-3 and intron 4-5. Such break will be repaired, and results in LoxP sites inserted into intron 2-3 and intron 4-5, respectively, by homologous recombination. When mating with Cre expression allele, sequence between two LoxP sites can be deleted in specific tissues or cells, so AdipoR1 gene will be disrupted by frameshift mutation (Supplementary Figure 1). The sequences of sgRNAs are 5′ ACGGCAGCACCTTTACTCAC 3′ and 5′ CTAGGCAAGCACACACTCGT 3′.

The CD4 Cre mice were purchased from the Model Animal Research Center of Nanjing University (from the Jackson Laboratory, United States). AdipoR1^fl/fl^CD4 Cre^±^ (CD4^Cre^AdipoR1^fl/fl^, KO) F2 mice were generated by crossing CD4 Cre mice with AdipoR1 lox mice. AdipoR1^+/+^CD4 Cre^±^ (CD4^Cre^AdipoR1^+/+^, WT) F2 mice were used as control mice.

All mice were housed according to specific pathogen-free grade animal feeding standards at an indoor temperature of 20–26°C and a 12-h day/night cycle. The mice were fed a standard diet after sterilization and had free access to food and water. All animals were euthanized for tissue collection. All experimental procedures abided by the guidelines of ethical regulations for institutional animal care and use in Nanjing Medical University and were approved by the Nanjing Medical University Ethics Committee for Animal Laboratory Research.

### AIA

Equal volumes of 2 mg/ml methylated bovine serum albumin (mBSA, Sigma-Aldrich), dissolved in ddH2O, and complete Freund’s adjuvant (Sigma-Aldrich) supplement were mixed and emulsified. In all experimental groups, except for the normal group, mice were immunized by subcutaneously injecting 100 μL of the mixture at days 0 and 14. On day 21, mice were immunized by injecting 10 μL of the emulsified mixture with 20 mg/ml mBSA into each side of the knee articular cavity. The mice were then monitored on daily basis by examiners blinded to the experimental design. As general physical signs, body weight and the diameter of the mediolateral knee joint were measured daily with an electronic scale and a vernier caliper, respectively.

### Histological Evaluation and Immunohistochemistry

Joint tissues were fixed in 10% (v/v) neutral buffered formalin, decalcified in a histological decalcifying agent, embedded in paraffin, and cut into sections 5 μm thick. The sections were stained with hematoxylin and eosin (H&E). Inflammation was scored using the following criteria: 0 = no inflammation; 1 = slight thickening of the lining or infiltration of some cells into the underlying layer; 2 = slight thickening of the lining with infiltration of some cells into the underlying layer; 3 = thickening of the lining, with an influx of cells into the underlying layer, and cells evident in the synovial space; and 4 = extensive infiltration of the synovium by inflammatory cells. For analysis of proteoglycans, 5 μm sections were stained with Safranin O-fast green and counterstained with hematoxylin. For quantification of the number of osteoclasts, total knee joint sections were stained for tartrate-resistant acid phosphatase (TRAP), using the Leukocyte Acid Phosphatase Kit (Sigma-Aldrich) according to the manufacturer’s protocol. The percentage of TRAP + cells present along the external bone surface was counted. Immunohistochemistry was performed using a Vectastain ABC kit. Tissues were stained with anti-TNF-α, anti-IL-1β, anti-IL-6 antibodies and an isotype control (Proteintech). Cells were counted visually at higher magnification by projection on a screen and cytokine-positive cells were identified by their brown color.

### Cell Preparation From Joints

Cell suspension from joints was prepared as described (12). To obtain a cell suspension from mouse joints, the entire legs were dissected, and the muscles and tendons were removed. To avoid contamination with the bone marrow, the femur was disarticulated by pulling the femoral head. The ligaments and tendons around the joints were cut a few millimeters. The knee and ankle joint were then opened. The legs were incubated in a mixture of enzymes containing 100 U/ml collagenase (Sigma-Aldrich) and 100 μg/ml DNaseI (Sigma-Aldrich) and shaken for 60 min at 37°C. After digestion, the legs were removed, and cells were purified using Percoll density centrifugation, filtered, and counted using automated cell counter.

### Cell Culture

Purified naïve T cells (CD4^+^CD62L^+^ T cells) from the spleen were isolated using the CD4^+^CD62L^+^ T Cell Isolation Kit II (Miltenyi) following the manufacturer’s instructions. The purity of the isolated naïve T cells was >95.5%. Naïve T cells were cultured in 96-well plates precoated in 10 μg/ml anti-CD3 mAb and 3 μg/ml anti-CD28 mAb, and then 3 ng/ml TGF-β (Peprotech), 40 ng/ml IL-6 (Peprotech), and then 30 ng/ml IL-23 (Peprotech) were added into the culture system for 72 h to induce Th17 differentiation. All cells were grown at 37°C with 5% CO_2_.

### Protein Extraction and Western Blot Analysis

An equal amount of protein (30 μg) was analyzed per sample using SDS polyacrylamide gel electrophoresis. Separated proteins were then transferred to a PVDF membrane. The membrane was incubated with primary antibodies overnight at 4°C with gentle shaking. The membranes were washed and incubated with a secondary antibody, followed by electrochemical luminescence detection. Relative protein levels were quantified by scanning densitometry and analyzed using ImageJ software. Protein detection was carried out using anti-GAPDH, anti-β-actin, anti-HIF-1α, anti-IL-17A, anti-GLUT1, anti-HK2 (proteintech) and anti-AdipoR1 (Abcam) Abs.

### Flow Cytometric Analysis

Single-cell suspensions from mouse spleen and joint tissues were prepared. A Zombie NIR Fixable Viability Kit (BioLegend) staining was performed for eliminating dead cells. Gated living cells were analyzed. Surface staining, such as anti-CD4 and isotype IgG control, was performed for 20–30 min. For intracellular staining, harvested cells were stimulated for 4–6 h in culture medium with PMA (Sigma-Aldrich), ionomycin (Sigma-Aldrich), and monensin (Sigma-Aldrich). Fixation/Permeabilization (eBioscience) was used for fixation and permeabilization, followed by Fc-R blocker (Miltenyi) and staining with the appropriate antibodies, including anti-IL-17A, anti-IFNγ, anti-Rorγt and their isotype IgG controls. Absolute cell numbers were calculated on the basis of the percentage of each cell population. All procedures were performed according to the manufacturer’s instructions.

### RNA Extraction and Real-Time PCR Analysis

Cells and joint tissues were collected for real-time PCR analysis. RNA samples were extracted using Trizol reagent (Takara) and RNA was converted to complementary deoxyribonucleic acid (cDNA) using the Prime Script^TM^ RT regent Kit following the manufacturer’s instructions (Takara). Real-time PCR analysis was performed using an ABI 7900 system (Applied BioSystems Inc) with the cycling parameters: 95°C for 10 min, followed by 40 cycles at 95°C for 15 s and 60°C for 1 min. Relative target gene expression was calculated as 2^–ΔΔCt^.

### RNA-Seq and Functional Pathway Analysis

RNA was isolated from activated splenic CD4^+^T cells from knockout (KO and WT mice. cDNA sequencing libraries were prepared using the TruePrep DNA Library Prep Kit V2 for Illumina and subjected to 2 × 150 paired-end sequencing. To identify differentially expressed genes (DEGs), fold expression changes were calculated for each gene by dividing the average fragments per kilobase of transcript per million mapped reads for the case by the average fragments per kilobase of transcript per million mapped reads for the control. The Gene Ontology and Kyoto Encyclopedia of Genes and Genomes analyses were used to identify cellular pathways and biological processes associated with DEGs, respectively.

### Statistical Analysis

Statistical analyses were performed using GraphPad 8.0. Data are expressed as the mean ± standard deviation. Statistical analysis was performed using one-way analysis of variance (ANOVA) tests and Student’ t-test. The values of *p* < 0.05 were considered significant (*p < 0.05, **p < 0.01, and ***p < 0.001).

## Results

### AdipoR1 Deficiency Reduces Th17 Cell Differentiation *in vitro*

**FIGURE 1:**
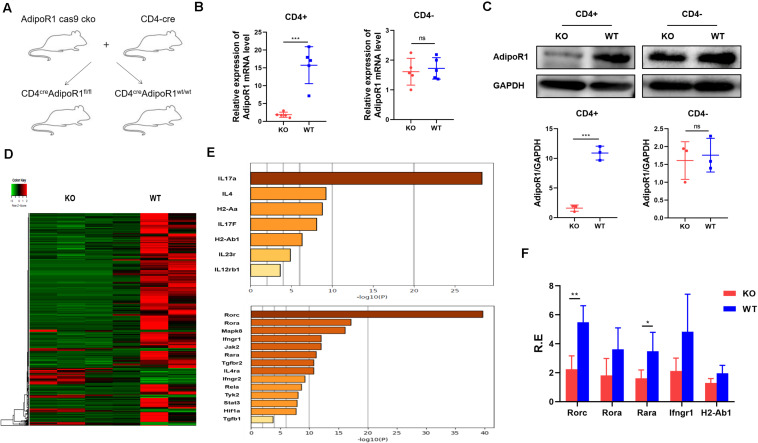
Transcriptome profiling of CD4^+^T cells in KO and WT mice. **(A)** T cell-specific AdipoR1 knockout (KO) mice were generated using the CD4-Cre/AdipoR1 flox system and their phenotypes analyzed. **(B)** Relative *AdipoR1* mRNA expression in CD4^+^ cells and CD4^–^ cells was measured by RT-PCR (*n* = 5). **(C)** AdipoR1 protein levels were measured by western blot (*n* = 3). **(D)** Heat map analysis of 646 significantly differentially expressed genes. Individual mouse samples are shown in columns and differentially expressed genes in rows. Red indicates increased expression. Green indicates decreased expression (*n* = 3). **(E)** Top genes and transcripts in the Th17 pathway. **(F)** RT-PCR assay to validate RNA-seq results (*n* = 4–5). All data are shown as mean ± SD (*p < 0.05, **p < 0.01, and ***p < 0.001).

We previously showed that AD could exacerbate collagen-induced arthritis progression by enhancing Th17 cell differentiation. To further clarify the role of the AD/AdipoR1 pathway in T cell response, we generated T cell-specific AdipoR1 KO mice by utilizing the CD4-Cre/AdipoR1 flox system (CD4^cre^ AdipoR1^fl/fl^) (Figure 1A). Only weak AdipoR1 expression can be observed in CD4^+^ T cells from KO mice, as compared with those in WT mice. However, there is no significant differences in AdipoR1 expression of CD4- T cells between KO and WT mice (Figures 1B,C). We compared T helper cell differentiation between KO and WT mice. The percentages and absolute numbers of CD4^+^ IFNγ^+^ Th1, CD4^+^ IL-4^+^ Th2, CD4^+^IL-17A^+^ Th17 and CD4^+^ CD25^+^ Foxp3^+^ Treg showed no significant differences between KO and WT group in healthy mice (Supplementary Figure 2). We analyzed the activated CD4^+^T cell gene expression profiles in both KO and wildtype (WT) mice using RNA sequencing (RNA-seq) and identified DEGs. We identified 646 DEGs in KO mice, including 551 down-regulated genes (Figure 1D). Multiple Th17 associated genes were markedly down-regulated in T cells from the KO group, and *Rorc* showed the strongest down-regulated signal (Figures 1E,F). To validate RNA-seq results, we performed RT-PCR for 18 selected targets involved in Th17 cell differentiation: *Rorc*, *Rora*, *Mapk8*, *Ifngr1*, *Jak2*, *Rara*, *Tgfbr2*, *IL4ra*, *Ifngr2*, *Tyk2*, *Stat3*, *IL-17A*, *IL-4*, *H2-Aa*, *IL-17F*, *H2-Ab1*, *IL-23R*, and *IL-12Rb1*. Consistent with our RNA-seq results, RT-PCR showed that *Rorc* expression was significantly lower in the KO group (Figure 1E). The top five down-regulated genes in KO mice were *Rorc*, *Rora*, *Rara*, *Ifngr1*, and *H2-Ab1*. These data support our previous findings that the AD/AdipoR1 pathway regulates Th17 cell associated gene expression.

**FIGURE 2:**
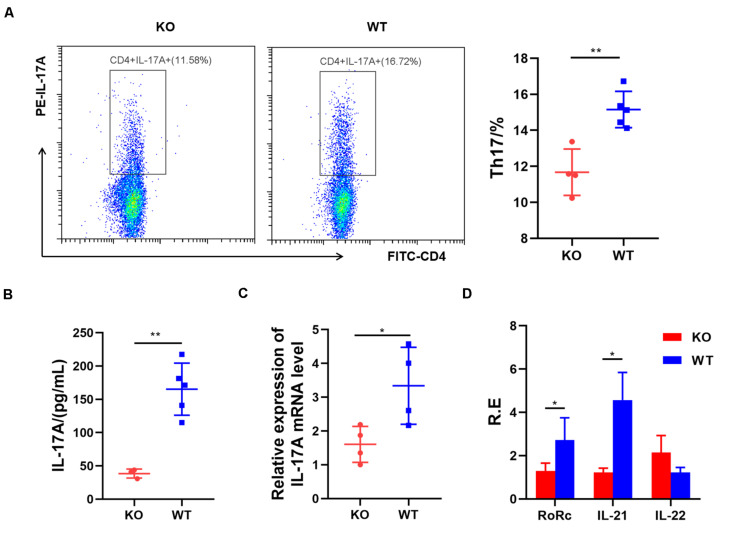
AdipoR1 knockout inhibits Th17 cell differentiation in culture. **(A)** Naïve T cells were cultured under Th17 polarization conditions. Representative flow cytometry plots of the CD4^+^IL-17A^+^ live cell populations were shown (*n* = 4–5). **(B)** Culture supernatants were analyzed for the expression of IL-17A by ELISA (*n* = 3–5). **(C)** Relative IL-17 related gene expression was measured using real-time PCR (*n* = 4). **(D)** Relative *Rorc*, *IL-21*, and *IL-22* mRNA expression was measured using real-time PCR (*n* = 4). All data are shown as mean ± SD (*p < 0.05, **p < 0.01).

Next, we examined the role of AdipoR1 in Th17 cell differentiation. Naïve splenic CD4^+^T cells from KO and WT mice were induced toward Th17 differentiation for 72 h. The Th17 cell frequency was reduced significantly in the KO group (*p* = 0.0026) (Figure 2A), compared with those in the WT group. Loss of AdipoR1 downregulated IL-17A production in Th17 cultures (*p* = 0.0017) (Figure 2B). IL-17 mRNA expression was approximately 50% lower in KO group CD4 + T cells during differentiation into Th17 than those observed in the WT group (*p* = 0.0329) (Figure 2C). We also detected the expression of other Th17 related genes, including *Rorc*, *IL-21*, and *IL-22*. Consistent with *IL-17* expression levels, mRNA levels of *Rorc* and *IL-21* markedly decreased in KO mice (*p* < 0.05). However, we failed to detect the changes of IL-22 expression *in vitro* (Figure 2D). These results demonstrate that blocking AdipoR1 can reduce Th17 differentiation *in vitro*.

### AdipoR1 Deficiency Ameliorates Disease Activity in AIA Mice

**FIGURE 3:**
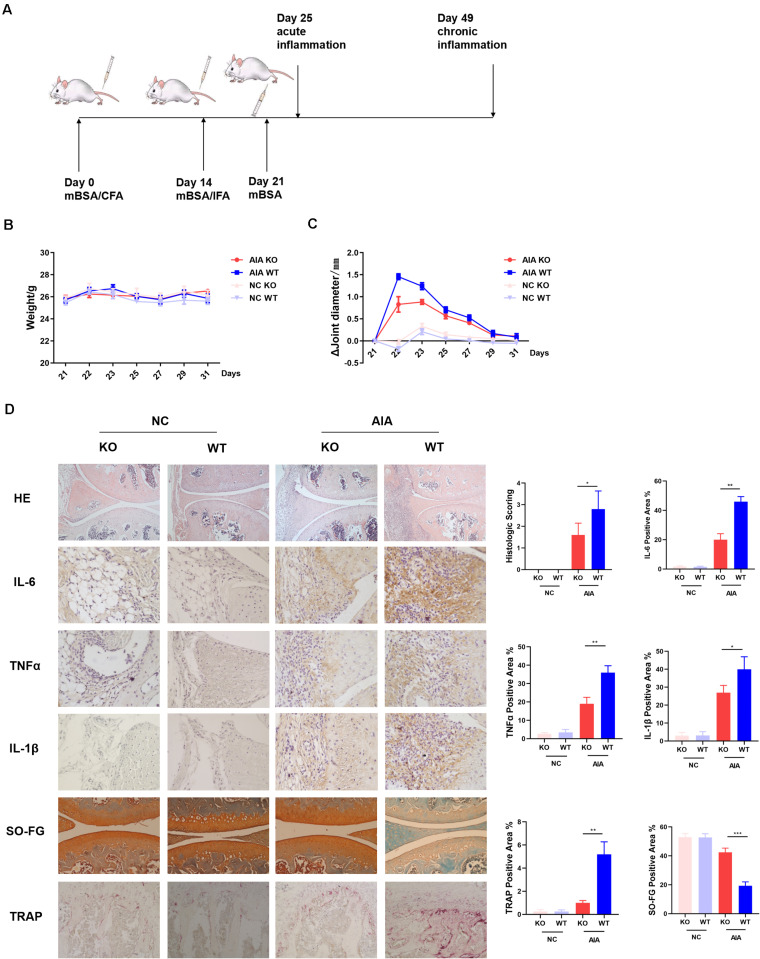
AdipoR1 knockout in T cells ameliorates antigen-induced arthritis (AIA) disease. **(A)** Timeline of AIA induction. **(B)** Weights were recorded daily after the 3rd immunization (*n* = 5). **(C)** Mean diameters of knee joints were recorded daily after the 3rd immunization (*n* = 5). **(D)** Photomicrographs for knee joint sections stained with H&E on day 25 (original magnification 100×) (*n* = 3–5). Photomicrographs of immunohistochemical staining of IL-6, TNFα and IL-1β in knee joint tissues on day 25, positive cells were stained with intense brown color (original magnification 400×) (*n* = 3). Knee joints were stained for TRAP positivity (left) on day 25 post AIA (original magnification 400×) and quantified by computer analysis (*n* = 3). Proteoglycan staining was demonstrated by safranin O-fast green immunostaining (original magnification 200×) (*n* = 3) (*p < 0.05, **p < 0.01, and ***p < 0.001).

Previously we had shown that AD could promote differentiation of naïve T cells into Th17 cells, resulting in enhanced synovitis and bone erosion in collagen-induced arthritis models (5). To further determine the role of AdipoR1 in the pathogenesis of autoimmune arthritis, we observed the incidence of arthritis in T cell-specific AdipoR1 KO AIA mice (Figure 3A). The arthritic mice did not exhibit a marked reduction in body weight (Figure 3B). Arthritic symptoms were examined by measurement of the diameter of knee joint swelling. The mean diameter of knee joints increased rapidly after the third immunization and reached its maximum at day 22 and then gradually declined in WT mice. However, the mean knee joint diameter was lower in KO mice than that in WT mice from day 22 (Figure 3C). As shown in Figure 3D, the knee joint from normal control group exhibited clear and complete histological architecture under the microscope, whereas the knee joints from AIA mice showed synovial hyperplasia and infiltration of massive inflammatory cells. The KO group preserved almost normal histological architecture of the knee joints with mild synovial hyperplasia, less inflammatory cells infiltration and less erosion of synovial tissues. The histological score remarkably decreased in KO group compared with WT group in AIA mice. Similarly, immunohistochemistry staining showed that IL-6, TNFα and IL-1β protein expression was diminished in KO group of AIA mice. Also, compared to WT, osteoclast numbers within the medullary space significantly reduced in KO group of AIA mice. Cartilage of KO mice in AIA models demonstrated less proteoglycan depletion as shown by Safranin-O staining (Figure 3D).

### AdipoR1 Deficiency Reduces Th17 Differentiation in AIA Mice

**FIGURE 4:**
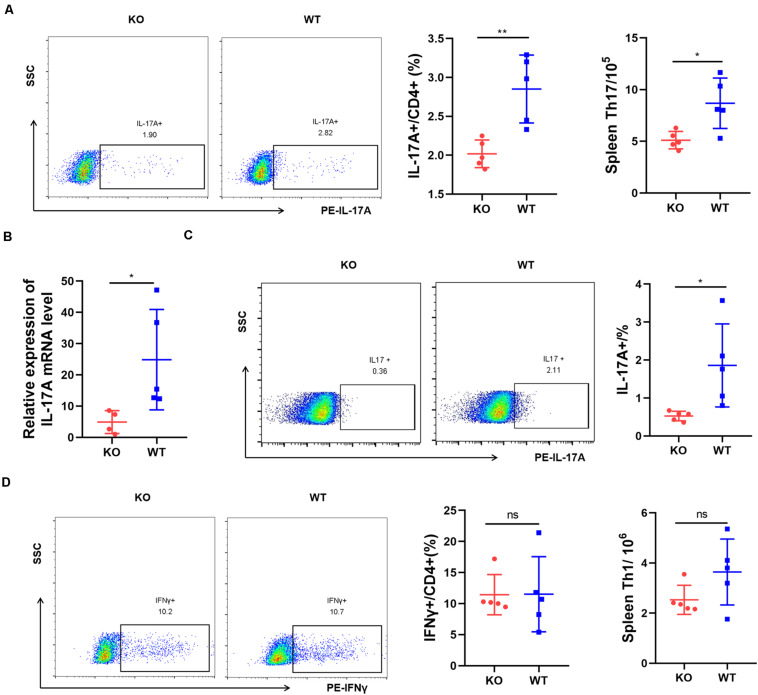
AdipoR1 knockout inhibits Th17 cell differentiation in antigen-induced arthritis (AIA). **(A)** Flow cytometric analysis of IL-17A expressions in CD4^+^ T cells isolated from spleen were shown (*n* = 5). **(B)** *IL-17A* mRNA expressions in joint tissues were measured by RT-PCR (*n* = 4–5). **(C)** Flow cytometric analysis of IL-17A expressions in lymphocytes isolated from joint tissues were shown (*n* = 5). **(D)** Flow cytometric analysis of IFNγ expressions in CD4^+^ T cells isolated from spleen were shown (*n* = 5). All data are shown as mean ± SD (*p < 0.05, **p < 0.01).

We tested whether AdipoR1 deficiency affects immune cell populations. Th17 cells, which preferentially produce IL-17A, play a key role in the inflammatory response in RA. We analyzed the expression of IL-17A in AIA mice. After immunization, flow cytometric analysis at day 25 indicated that splenic CD4 + IL-17A + Th17 cells from KO AIA mice was half than those observed in the WT group (*p* = 0.0043) (Figure 4A). Moreover, there was an approximate threefold reduction in IL-17A expression in joint tissue from KO mice (*p* = 0.0268) (Figures 4B,C). No significant changes in Th1 cell numbers were observed (Figure 4D). We analyzed the expression of other proinflammatory cytokines in joint tissues using real-time PCR and western-blotting. Expression of IL-6, TNFα, and IL-1β were significantly down-regulated in joint tissue of KO mice (Supplementary Figure 3). Together, our data suggest that AdipoR1 knockout ameliorates arthritis symptoms in AIA mice, and is accompanied by decreased IL-17 expression.

### Inhibition of AdipoR1 Reduces Glycolysis in Th17-Polarized Cells by Reducing HIF-1α Protein Levels

**FIGURE 5:**
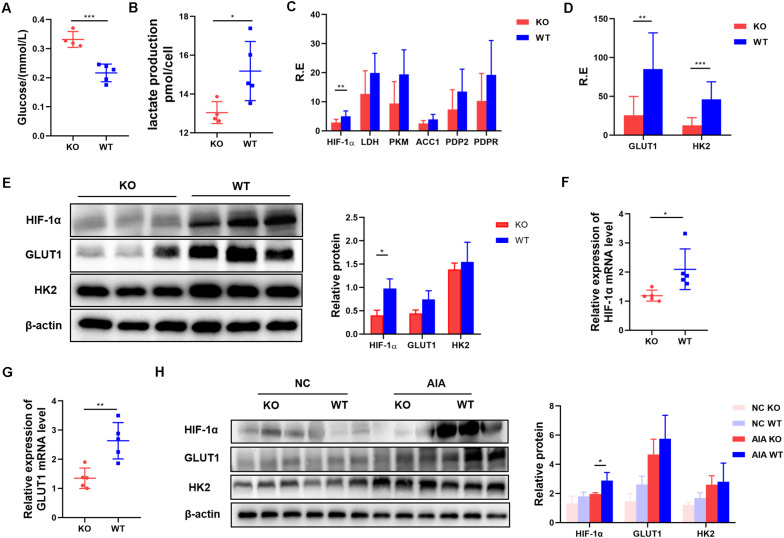
Inhibition of AdipoR1 reduces glycolysis in Th17-polarized cells by reducing HIF-1α protein levels. **(A,B)** Naïve T cells were cultured under Th17 polarization conditions. Culture medium was collected to measure glucose and lactate production (*n* = 4–5). **(C)** Relative *HIF-1*α, *LDH*, *PKM*, *ACC1, PDP2* and *PDPR* mRNA expression were measured using RT-PCR (pooled data from *n* = 2 experiments, 4–5 mice each). **(D)** Relative *GLTU1* and *HK2* mRNA expression was measured by RT-PCR (*n* = 4–5). **(E)** HIF-1α, GLUT1, and HK2 protein levels were measured by western blot (*n* = 3). **(F)** Relative *HIF-1*α mRNA expression in joint tissues of AIA models was measured by RT-PCR (*n* = 5). **(G)** Relative *GLUT1* mRNA expression in joint tissues of AIA models was measured by RT-PCR (*n* = 5). **(H)** HIF-1α, GLUT1, and HK2 protein levels in joint tissues of AIA models were measured by western blot (*n* = 3). All data are shown as mean ± SD (*p < 0.05, **p < 0.01, ***p < 0.001).

Our data suggest that AdipoR1 can affect Th17 differentiation by regulating Th17 related transcription factors and gene expression. AdipoR1 is reported to be a regulator involved in glucose metabolism, so we tested the glucose levels in the supernatants of naïve T cells pushed toward Th17 differentiation. We found that glucose utilization was reduced in the KO group (*p* = 0.0004) (Figure 5A). Th17 cells rely on glycolysis for energy and for substrates for proliferation and differentiation. To compare glycolytic flux in KO and WT groups under Th17 polarization, we quantified lactate production in naïve T cells 72 h after stimulation. T cells from the KO group produced less lactate than those from the WT group did (*p* = 0.0357) (Figure 5B).

To explore whether AdipoR1 KO has an intrinsic defect in glycolysis during Th17 differentiation, we measured the expression of six major glycolysis-related genes (*HIF-1*α, *LDH, PKM, ACC1, PDP2*, and *PDPR*) in activated naïve T cells. Only *HIF-1*α expression was decreased in the KO group (*p* = 0.0065) (Figure 5C). Expression of HIF-1α-mediated-glycolysis genes, including *GLUT1* and *HK2*, also decreased in the KO group (*p* < 0.05) (Figures 5D,E). Expression of HIF-1α and metabolic rate-limiting enzymes that downstream of HIF-1α was significantly down-regulated in the joint tissue of KO mice (*p* < 0.05) (Figures 5F–H).

### VH298 Suppresses the Effect of AdipoR1 Inhibition

**FIGURE 6:**
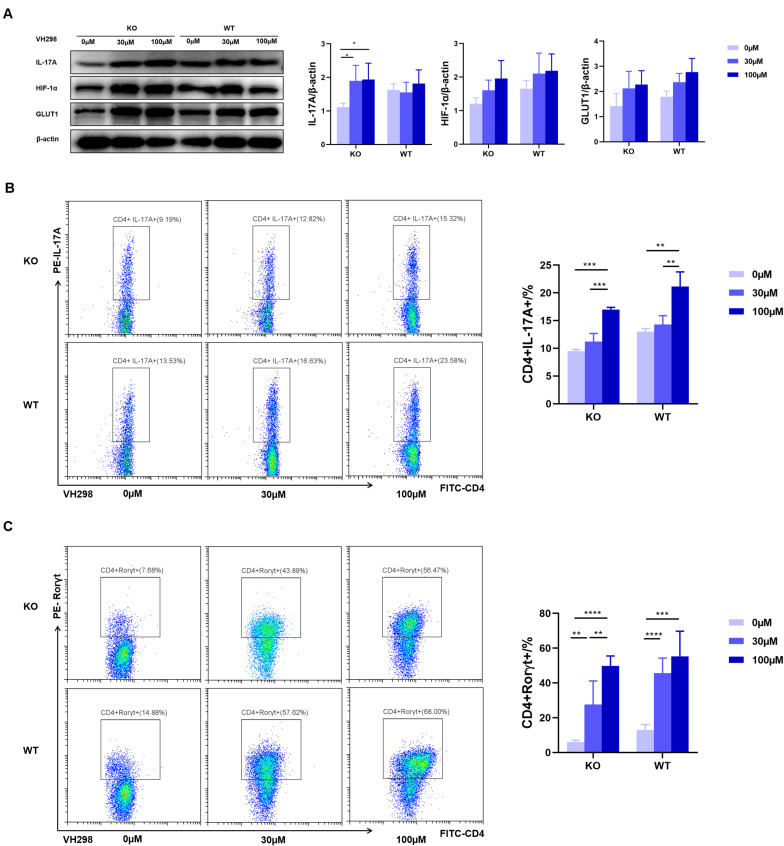
VH298 suppresses the effects of AdipoR1 knockout. Naïve CD4^+^ T cells were cultured under Th17-polarizing conditions. Cells were cultured in the presence of VH298 (0, 30 and 100 μM) for 72 h. **(A)** IL-17A, HIF-1α, and GLUT1 protein levels were measured by western blot (pooled data from *n* = 3 experiments, 3 mice each). **(B)** Representative flow cytometry plots of the CD4^+^IL-17A^+^ live cell populations were shown (*n* = 3–5). **(C)** Representative flow cytometry plots of the CD4^+^ Rorγt^+^ live cell populations were shown (*n* = 3–5). All data are shown as mean ± SD (*p < 0.05, **p < 0.01, ***p < 0.001).

To test whether AdipoR1-mediated Th17 differentiation through regulation of HIF-1α expression, we used VH298, a new compound that could upregulate HIF-target genes expression at both mRNA and protein levels. Under Th17 polarization, and compared with the WT group, we observed an approximately twofold reduction in the frequency of Th17 cells in the KO group. VH298 increased the HIF-1α protein level in both the KO and WT groups and synchronously up-regulated IL-17 and GLUT1 expression in Th17 cells (Figure 6A). As expected, accumulation of HIF-1α partially restored Th17 cell differentiation in KO mice (Figure 6B). Moreover, expression of Rorγt also significantly increased upon VH298 stimulation (Figure 6C). Taken together, our data suggest that AdipoR1 regulates Th17 cell differentiation at least in part through modulating the HIF-1α-mediated glucose metabolism pathway.

## Discussion

Increasing evidence shows that, by acting on its receptors, AD plays an important role in regulating immune and inflammatory processes (13). Our previous studies show that AD and AdiopR1 are highly expressed in RA synovial tissues (4). We also showed, for the first time, that AD plays an important role in promoting Th17 cell differentiation (5). We extended our previous studies and revealed that AdipoR1 regulates Th17 cell differentiation at least in part by modulating HIF-1α-mediated glycolysis in T cells. Our data highlight the relevance of AdipoR1 as a potential novel therapeutic target in RA.

The Th17 cell subset, which produces IL-17, is involved in the pathogenesis of RA with regard to synovial hypertrophy, enhanced osteoclastogenesis, and neoangiogenesis (14, 15). Loss of AdipoR1 inhibited Th17 differentiation and reduced the production of IL-17. In our study, RNA-seq analysis identified seven genes and 14 transcription factors in the Th17 pathway that were significantly down-regulated in the KO group. To validate the RNA-seq results, we performed RT-PCR assays and identified *Rorc* (retinoic acid receptor-related orphan receptor gamma, Rorγ) as the most DEG. *Ror*γ*t* is a splicing variant of *Rorc*, and is mainly expressed in CD4^+^CD8^+^ thymocytes. Rorγt induces *IL-17* and *IL-17F* transcription in naïve CD4^+^T helper cells. Moreover, Rorγt is required for *IL-17* and *IL-17F* expression in response to IL-6 and TGF-β, the IL-17 inducing cytokines (16). Our data show that AdipoR1 can promote Th17 cell differentiation through upregulating *Rorc* expression. Moreover, AdipoR1 KO inhibits the expression of IL-21, an essential autocrine amplification factor for Th17 cells induction (17, 18).

We also showed that AdipoR1 regulates HIF-1α-mediated glycolysis during Th17 cell differentiation. In the pathogenesis of RA, T cells start to proliferate and differentiate when stimulated. Metabolism reprogramming is crucial for meeting energy requirements and providing various substrates which are indispensable for T cell proliferation and differentiation (19). After stimulation, CD4^+^ naïve T cells become highly proliferative and differentiate into Th cells. Then glycolysis, oxidative phosphorylation, the pentose phosphate pathway, the hexosamine pathway, and fatty acid metabolism become active. Subtle differences in these metabolic programs will lead to CD4 + naïve T cells differentiation into different T helper cell lineages (20–22). Th17 cells rely more on aerobic glycolysis (23). AdipoR1 is a cell metabolism regulator in cancer (24–26). Lin-Yu Sun et al. reported that AdipoR1 is involved in the glucose metabolism in acute myeloid leukemia (27). Based on these reports, we hypothesized that inhibition of AdipoR1 may also affect glycolysis during Th17 cell differentiation. Evaluation of glucose utilization and lactate production confirmed our hypothesis.

The mTOR-HIF-1α/myc-glycolysis axis plays a critical role in the metabolism control in Th17 cells (28). HIF-1α is a major regulator of cellular metabolism and is a key transcription factor, orchestrating the expression of glycolytic enzymes (29). Our data show that glycolysis is reduced and Th17 cell proliferation and cytokine production are inhibited in the presence AdipoR1 KO mice. We tested the key genes of glycolysis in Th17 differentiation and found that *HIF-1*α expression was down-regulated in KO mice *in vivo* and *in vitro*. Additionally, the expression of key glycolysis rate-limiting enzymes in the HIF-1α pathway was also reduced in KO mice.

HIF-1α protein is unstable and rapidly degraded via the ubiquitin-proteasome pathway through an E3 ubiquitin ligase (30). The von Hippel-Lindau is the recognition component of the E3-ubiquitin ligase complex (31). Frost et al. identified a von Hippel-Lindau inhibitor named VH298, which caused selective on-target accumulation of hydroxylated HIF-1α and upregulation of HIF-target genes at both mRNA and protein levels (32). VH298 inhibits HIF-1α degradation, leading to HIF-1α accumulation. Rorγt expression and Th17 cell frequencies significantly increased with VH298 treatment in a dose dependent manner under Th17 polarization. Our data suggest that HIF-1α accumulation could partially, but not completely, restore the ability of AdipoR1-deficent CD4^+^T cells to differentiate into Th17 cells. Therefore, AdipoR1 may also regulate Th17 differentiation through other pathways.

## Conclusion

This research presents the first report of AdipoR1 functioning as a regulator to promote HIF-1α-mediated glycolysis and to potentiate Th17 differentiation in RA. Our data suggest that AdipoR1 could be considered as a novel therapeutic target for autoimmune arthritis.

## Data Availability Statement

The raw data supporting the conclusions of this article will be made available by the authors, without undue reservation.

## Ethics Statement

The animal study was reviewed and approved by Institutional Animal Care and Use Committee of Nanjing Medical University (Permit Number: IACUC-2013090101).

## Author Contributions

MZ, WT, and QZ contributed to study conception, design, analysis and interpretation of data, and wrote the manuscript. QZ, LW, JJ, SL, AL, and PZ contributed to acquisition of data. All authors reviewed the manuscript before submission.

## Conflict of Interest

The authors declare that the research was conducted in the absence of any commercial or financial relationships that could be construed as a potential conflict of interest.
