# Supplementary material for: Critical Role of AdipoR1 in Regulating Th17 Cell Differentiation Through Modulation of HIF-1α-Dependent Glycolysis
Source: Front Immunol. 2020 Aug 18;11:2040. doi: 10.3389/fimmu.2020.02040 (PMC7461876; doi:10.3389/fimmu.2020.02040)

**Supplementary Figure 1**

Strategic design and final vector sequencing results. (A) Schematic diagram of the AdipoR1 gene knockout targeting vector. (B) The sequencing results at Exon 3-4 and LoxP, while Exon 3-4 is marked with blue, the loxp is marked with red.


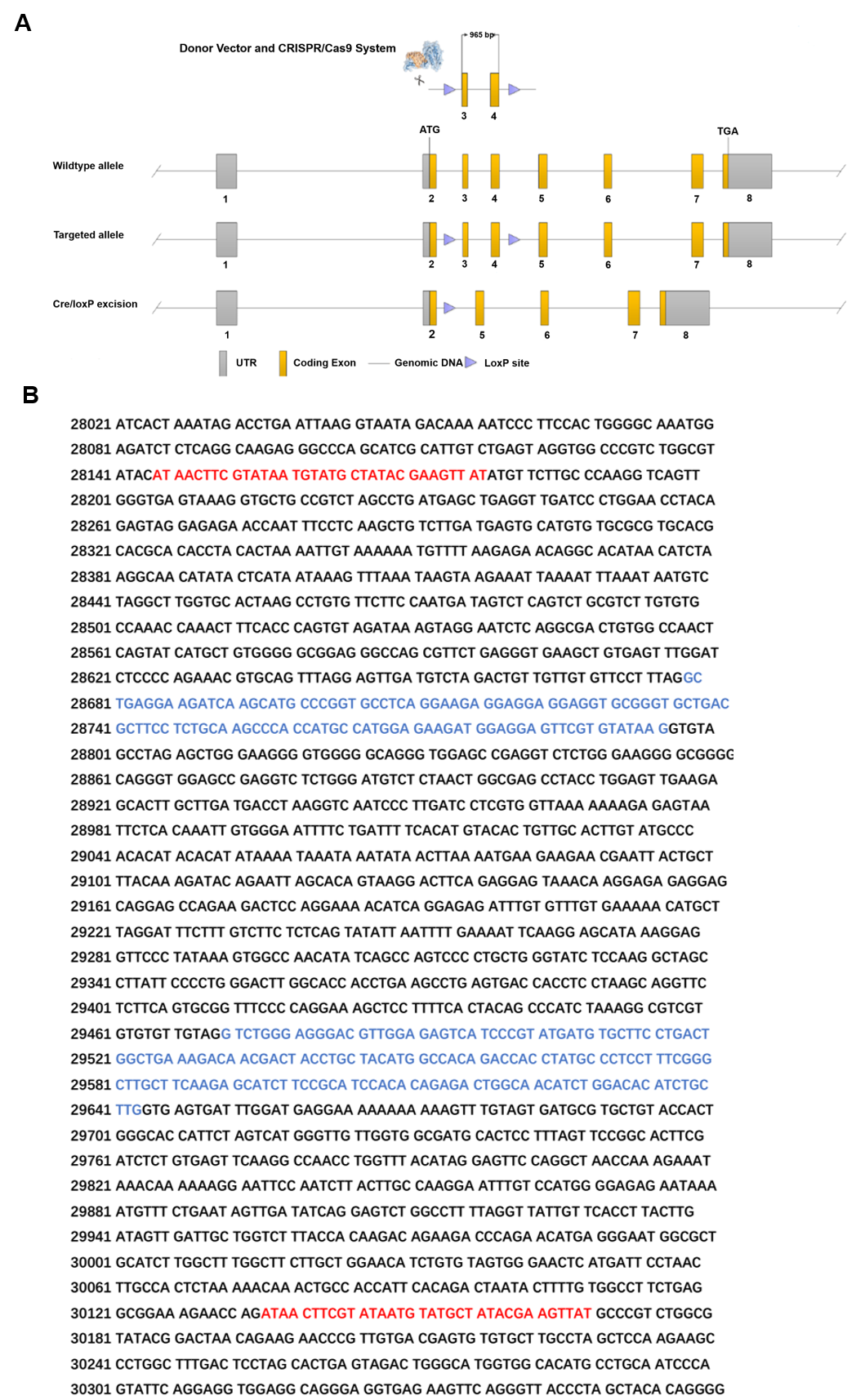


**Supplementary Figure 2**

No significant differences in T cell subsets under physiological conditions. Percentages and absolute cell numbers of CD4^+^IFNγ^+^, CD4^+^IL-4^+^, CD4^+^IL-17A^+^ and CD4^+^CD25^+^Foxp3^+^ cells in spleen were measured by flow cytometry in 6–8-week-old male mice (n = 3-5). All data were shown as mean ± SD.


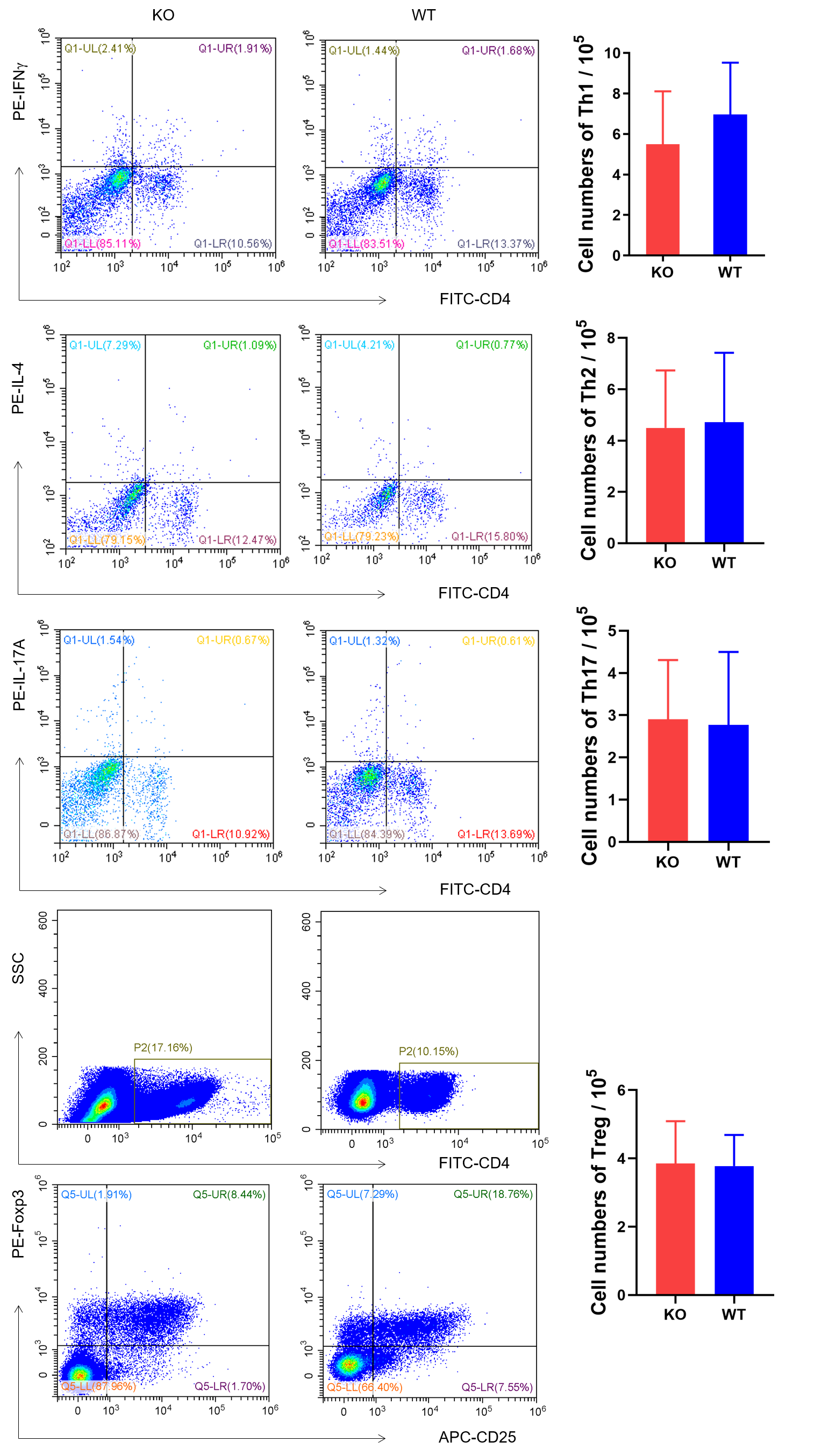


**Supplementary Figure 3**

Knockout of AdipoR1 reduces the expression of inflammatory factors in joint tissue. (A) Relative expressions of IL6, TNFα, IL1β mRNA were measured by RT-PCR (pooled data from n=3 experiments, 4-5 mice each). (B) Protein levels of IL6, TNFα, IL1β mRNA were measured by western blot (pooled data from n=2 experiments, 3-5 mice each). All data were shown as mean ± SD (**p* < 0.05, ***p* < 0.01, ****p* < 0.001)


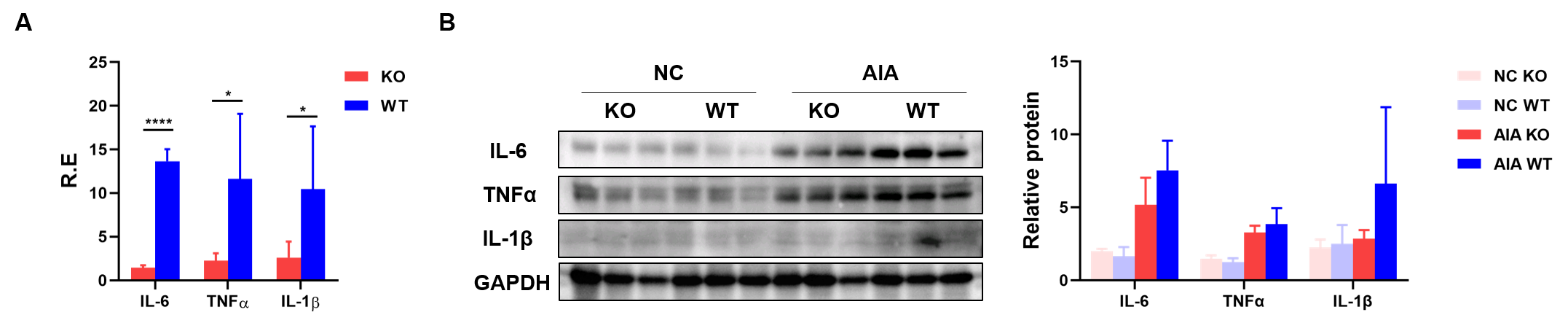

Supplement: Supplementary file 1 [file Data_Sheet_1.doc]
